# 探讨PET/CT原发灶SUVmax在肺鳞癌患者术后预后中的意义及与临床病理特征的关系

**DOI:** 10.3779/j.issn.1009-3419.2016.04.03

**Published:** 2016-04-20

**Authors:** 红亮 任, 文贵 徐, 健 尤, 秀宇 宋, 慧 黄, 宁 赵, 秀宝 任, 新伟 张

**Affiliations:** 1 300060 天津，天津医科大学肿瘤医院生物治疗科，国家肿瘤临床医学研究中心，天津市肿瘤防治重点实验室 Department of Biotherapy, Tianjin Medical University Cancer Institute and Hospital, National Clinical Research Center of Cancer, Key Laboratory of Cancer Prevention and Therapy of Tianjin, Tianjin 300060, China; 2 300060 天津，天津医科大学肿瘤医院生物治疗科，国家肿瘤临床医学研究中心，分子影像与核医学诊疗科 Department of Molecular Imaging and Nuclear Medicine, Tianjin Medical University Cancer Institute and Hospital, Tianjin 300060, China; 3 300060 天津，天津医科大学肿瘤医院生物治疗科，国家肿瘤临床医学研究中心，肺部肿瘤科 Department of Lung Cancer, Tianjin Medical University Cancer Institute and Hospital, Tianjin 300060, China

**Keywords:** PET/CT, 肺鳞癌, 最大标准化摄取值, 预后, PET/CT, Lung squamous cell carcinoma, SUVmax, Prognosis

## Abstract

**背景与目的:**

肺癌居于全球男性及女性癌症相关死亡原因的首位，大多数患者在确诊时已属晚期，5年生存率仅为18%。肺癌可分为非小细胞肺癌（non-small cell lung carcinoma, NSCLC）和小细胞癌（small cell lung carcinoma, SCLC），其中NSCLC占肺癌的80%-85%，NSCLC根据组织学可主要分为腺癌（约占40%），鳞状细胞癌（20%-30%）和大细胞癌（10%），针对驱动基因的靶向治疗在肺腺癌中取得一定成绩，但在肺鳞癌的治疗中收效甚微，肺鳞癌的诊治更需得到关注，^18^F-脱氧葡萄糖（fluorodeoxyglucose, FDG）正电子发射断层扫描/计算机体层摄影（positron emission tomography/computed tomography, PET/CT）越来越多地应用于肺癌的诊断与分期中，本研究旨在探讨^18^F-FDG PET/CT原发灶最大标准摄取值（maximum standardized uptake value, SUVmax）在肺鳞癌患者术后预后中的意义及与临床病理特征的关系。

**方法:**

回顾分析2005年5月-2014年10月收治的182例初治、接受PET/CT检查、行根治术的原发肺鳞癌患者的临床影像病理及随访资料。采用*Kaplan-Meier*法及*Cox*模型分析患者生存情况，并分析原发灶SUVmax与各临床病理因素的关系。

**结果:**

182例肺鳞癌患者原发灶SUVmax以13.0为界分为两组，SUVmax > 13.0组与≤13.0组患者的中位总生存期分别为56个月和87个月，差异具有统计学意义（*P*=0.022）。原发灶SUVmax与性别、肿瘤最大径、肿瘤-淋巴结-转移（tumor-node-metastasis, TNM）分期、中性粒细胞、中性粒细胞/淋巴细胞比例（neutrophil-lymphocyte ratio, NLR）存在正相关性，与血红蛋白呈负相关（*P* < 0.05）。*Cox*多因素分析显示SUVmax（HR=1.714, 95%CI: 1.021-2.876, *P*=0.042）、TNM分期（HR=1.677, 95%CI: 1.231-2.284, *P*=0.001）均为患者生存的独立预后影响因子，提示SUVmax有独立于病理TNM分期之外的预后价值。而且，SUVmax在Ⅰ期肺鳞癌患者的预后中有意义（*P*=0.045）。

**结论:**

PET/CT SUVmax对肺鳞癌患者术后生存的预测有重要的价值，是独立于TNM分期之外的一个重要预后因素，并且原发灶SUVmax与多个临床病理因素间存在相关性。

## Abstract (en)

**Background and objective:**

Lung cancer is the leading cause of cancer death in men and women in the world, more than one-half of cases are diagnosed at a advanced stage, and the overall 5-year survival rate for lung cancer is 18%. Lung cancer is divided into non-small cell lung carcinoma (NSCLC) and small cell lung carcinoma (SCLC). Approximately 80%-85% of cases are NSCLC which includes three main types: adenocarcinoma (40%), squamous cell carcinoma (SCC) (20%-30%), and large cell carcinoma (10%). Although therapies that target driver mutations in adenocarcinomas are showing some promise, they are proving ineffective in smoking-related SCC. We need pay more attention to the diagnosis and treatment of SCC. ^18^F-FDG positron emission tomography (PET)/computed tomography (CT) has emerged as an accurate staging modality in lung cancer diagnosis. The aim of this study is to investigate the role of maximum standardized uptake value (SUVmax) on PET-CT in prognosis and its correlation with clinicopathological characteristics in resectable SCC.

**Methods:**

One hundred and eighty-two resectable SCC patients who underwent PET/CT imaging between May 2005 and October 2014 were enrolled into this retrospectively study. All the enrolled patients had underwent pulmonary resection with mediastinal lymph node dissection without preoperative chemotherapy or radiotherapy. Survival outcomes were analyzed using the *Kaplan-Meier* method and multivariate *Cox* proportional hazards model. Correlation between SUVmax and clinicopathological factors was analysed using *Pearson* correlation analysis and *Spearman* rank correlation analysis.

**Results:**

The patients were divided into two groups on the basis of SUVmax 13.0 as cutoff value, and patients with SUVmax more than 13.0 had shorter median overall survival than patients less than 13.0 in univariate analysis (56 months *vs* 87 months; *P*=0.022). There was remarkable correlation between SUVmax and gender, tumor size, tumor-node-metastasis (TNM) stage, neutrophil, NLR, hemoglobin (*P* < 0.05). Multivariate *Cox* analysis demonstrated that SUVmax (HR=1.714, 95%CI: 1.021-2.876, *P*=0.042), TNM stage (HR=1.677, 95%CI: 1.231-2.284, *P*=0.001) were independent predictors for survival. Furthermore, univariate survival analysis showed significant difference by SUVmax in patients of stage Ⅰ (*P*=0.045).

**Conclusion:**

SUVmax may be of importance prognostic factor independent of TNM stage, which was considerable for risk stratification in patients with TNM stage. Besides, there was correlation between SUVmax of primary tumor and clinicopathological characteristics.

肺癌居于全球男性及女性癌症相关死亡原因的首位^[1]^。2015年美国肺癌新诊断病例221, 200例（男性115, 610例，女性105, 590例），肺癌相关死亡病例158, 040例（男性86, 380例，女性71, 660例），大多数患者在确诊时已属晚期，5年生存率仅为18%^[2]^。肺癌可分为非小细胞肺癌（non-small cell lung carcinoma, NSCLC）和小细胞癌（small cell lung carcinoma, SCLC），其中NSCLC约占肺癌的80%-85%，NSCLC根据组织学可分为腺癌（约占40%），鳞状细胞癌（21%-30%）和大细胞癌（10%）等亚型，针对驱动基因的靶向药物在肺腺癌的治疗中取得一定成绩，但在肺鳞癌的治疗中收效甚微^[3]^，肺鳞癌的诊治更需得到关注。精确分期对包括肺鳞癌在内的NSCLC规范化治疗方案的选择及预后判断具有重要的意义。^18^F-脱氧葡萄糖（fluorodeoxyglucose, FDG）正电子发射断层扫描/计算机体层摄影（positron emission tomography/computed tomography, PET/CT）实现了解剖结构和代谢功能两种图像的融合，已成为NSCLC分期的重要诊断手段^[4, 5]^。最大标准化摄取值（maximum standardized uptake value, SUVmax）是目前常用的半定量诊断指标，研究已知NSCLC原发肿瘤病灶的SUVmax与疾病的分期、淋巴结状态、组织学类型、肿瘤分化程度和肿瘤进展的速率相关联^[6]^。另外，高SUVmax已成为NSCLC患者不良预后因子之一^[7-9]^，但对于肺鳞癌患者的预后影响尚不清楚，本研究旨在探讨肺鳞癌患者术前PET/CT原发灶SUVmax与其临床病理特征及术后预后的关系。

## 1 材料与方法

### 1.1 一般资料

回顾性分析2005年5月-2014年10月于天津医科大学肿瘤医院就诊行肺部手术并经病理确诊的182例肺鳞癌患者。纳入标准：均为有完整的临床、病理及PET-CT影像检查资料的首诊患者，即此前未接受包括放疗、化疗、靶向治疗在内的任何干预性治疗；经完善的术前检查，并于术前接受PET/CT检查（行PET/CT检查至手术间隔5天-34天，中位12天），经评估可耐受手术者。排除标准：合并其他恶性肿瘤、终末期肝肾功能不全、糖尿病患者血糖控制不佳者。患者年龄为43岁-82岁，平均年龄65岁。

### 1.2 PET/CT检查

患者PET/CT检查使用美国GE公司生产的DiscoveryST4 PET/CT扫描仪，显像剂^18^F-FDG由天津医科大学肿瘤医院分子影像及核医学诊疗科提供，为pH值5-7，放射化学纯度≥95%的等渗溶液。患者检查当日空腹6 h以上，检查前测空腹血糖 < 6.8 mmol/L，经肘前静脉注射显影剂^18^F-FDG，剂量为3.7 MBq/kg-4.81 MBq/kg，平静休息60 min排尿后行全身PET及CT断层显像，显像范围为颅底部至股骨中段，PET图像行衰减校正及迭代法重建，PET、CT图像行多层面、多幅显示。所有图像均由3位经验丰富的核医学医师采用互盲法阅片，在放射性核素浓聚灶部位设置感兴趣区（region of interest, ROI），由计算机软件计算出ROI的SUVmax。肿瘤大小以CT肺窗上测量的最大径表示。

### 1.3 术式及术后病理

182例肺鳞癌患者中164例（90.1%）行肺叶切除及楔形切除术，15例（8.2%）行左全肺切除术，3例（1.7%）行右全肺切除；患者均进行同侧纵隔淋巴结清扫术。组织学高分化11例（6.1%），中分化114例（62.6%），低分化57例（31.3%）；50例（27.5%）患者出现肺门或纵隔淋巴结转移；临床分期参照第7版国际肺癌肿瘤-淋巴结-转移（tumor-node-metastasis, TNM）分期：Ⅰa期54例，Ⅰb期36例，Ⅱa期36例，Ⅱb期14例，Ⅲa期42例。

### 1.4 术后治疗及随访

患者信息通过其术后就诊检查记录、信访、电话随访所得，随访日期截止至2015年8月30日，随访时间为6个月-107个月，中位随访时间为38个月，至随访结束62例（34.1%）患者死亡。总生存期（overall survival, OS）指手术日开始至患者死亡或末次随访的时间。114例（62.6%）患者术后进行化疗、放疗或生物治疗，其中4例手术断端病理阳性者，1例术后首选放疗，其他3例均化疗。

### 1.5 统计学方法

使用统计学软件SPSS 21.0进行统计学分析，根据变量是否服从正态分布分别采用*Pearson*相关分析和*Spearman*等级相关分析；单因素生存分析采用*Kaplan-Meier*分析法，对影响预后的多因素分析采用*Cox*风险回归模型，*P* < 0.05为差异具有统计学意义。

## 2 结果

### 2.1 原发灶SUVmax与肺鳞癌临床病理因素间的相关性分析

肺鳞癌原发灶SUVmax与肿瘤大小、中性粒细胞、中性粒细胞/淋巴细胞比例（neutrophil-lymphocyte ratio, NLR）、血红蛋白（hemoglobin, HGB）之间采用*Pearson*相关分析，相关系数有统计学意义（*P* < 0.001、0.021、0.006、0.004），除血红蛋白外均呈正相关（*r* > 0），提示肺鳞癌原发病灶SUVmax随肿瘤大小、中性粒细胞、NLR的增长呈升高趋势，而随着HGB的增高呈降低趋势；SUVmax与性别、TNM分期之间的相关性分析采用*Spearman*等级相关分析，相关系数有统计学意义（*P*=0.012, *P*=0.022），提示原发灶SUVmax与性别和分期之间存在相关性（表 1）。

**1:** 肺鳞癌SUVmax与临床因素间的相关性分析 The relationship between SUVmax and clinicopathological characteristics

Variables	*r*	*P*	Association
Gender	0.186	0.012	Positive correlation
Age	0.076	0.308	No correlation△
Differentiation	0.050	0.617	No correlation
Tumor size	0.314	< 0.001	Positive correlation△
Lymph node metastasis	0.096	0.199	No correlation
TNM stage	0.170	0.022	Positive correlation
Neutrophil	0.171	0.021	Positive correlation△
Lymphocyte	-0.100	0.181	No correlation△
NLR	0.202	0.006	Positive correlation△
HGB	-0.212	0.004	Negative correlation△
△: *Pearson* correlation analysis; The others: *Spearman* rank correlation analysis; TNM: tumor-node-metastasis; NLR: neutrophil-lymphocyte ratio; HGB: hemoglobin.			

### 2.2 全组患者的生存分析

182例肺鳞癌患者原发灶SUVmax最小值2.7，最大值38.5，中位数13.0，以SUVmax的中位数13.0为界点分为≤13.0及 > 13.0两组，*Kaplan-Meier*生存分析表明，患者术前PET/CT SUVmax > 13.0组中位生存期短于≤13.0组（56个月*vs* 87个月），差异具有统计学意义（*P*=0.022，图 1）。对肺鳞癌患者预后相关的其他临床病理因素进行单因素分析，结果表明原发灶大小、TNM分期、中性粒细胞绝对值、体重指数（body mass index, BMI）、癌胚抗原（carcinoembryonic antigen, CEA）为患者总生存期的影响因素（*P* < 0.05，表 2）。将单因素分析结果中有意义的因素纳入*Cox*多因素分析显示，SUVmax（HR=1.714, 95%CI: 1.021-2.876, *P*=0.042）、TNM分期(HR=1.677, 95%CI: 1.231-2.284, *P*=0.001)均为患者总生存的独立预后影响因子，提示SUVmax有独立于病理TNM分期之外的预后价值。

**1:**
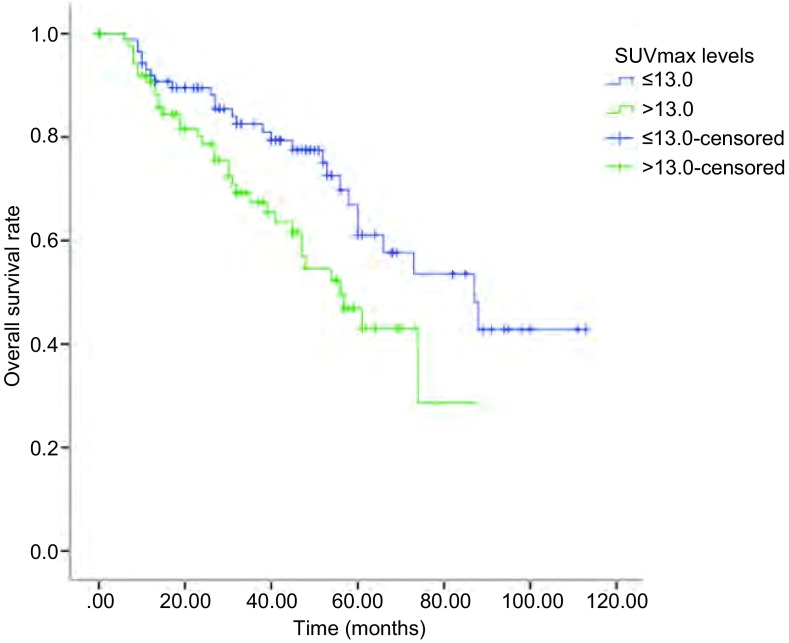
PET/CT原发灶SUVmax分组与182例肺鳞癌患者预后的关系 Relationship between SUVmax levels and prognosis of lung squamous cell carcinoma. PET/CT: positron emission tomography/computed tomography.

**2:** 肺鳞癌患者单因素生存分析 Univariate survival analysis of clinicopathological characteristics and prognosis

Variables	*n*	Median OS	*χ*^2^	*P*
Gender			2.262	0.133
Male	158	73		
Female	24	58		
Age (yr)			0.768	0.381
≤65	92	87		
> 65	90	60		
BMI				
≤22	45	56		
> 22	137	87	6.940	0.008
Tumor location			1.317	0.251
Central type	39	60		
Peripheral type	143	73		
Differentiation			0.115	0.944
Well	11	73		
Moderately	114	66		
Poorly	57	60		
Tumor size (cm)			4.569	0.033
≤3	68	88		
> 3	114	66		
TNM stage			12.471	0.002
Ⅰ	90	88		
Ⅱ	50	61		
Ⅲ	42	38		
Adjuvant therapy			2.043	0.153
No	68	74		
Yes	114	66		
Neutrophil (×10^9^/L)			4.802	0.028
≤4.4	97	87		
> 4.4	85	60		
Lymphocyte (×10^9^/L)			0.693	0.405
≤1.95	93	66		
> 1.95	89	73		
NLR			0.295	0.587
≤2.26	92	73		
> 2.26	90	74		
SCC (*μ*g/L)			1.773	0.183
≤1.5	119	73		
CEA (*μ*g/L)				
≤5	137	88	5.429	0.020
> 1.5	63	74		
> 13.0	90	56		
SUVmax			5.210	0.022
≤13.0	92	87		
> 13.0	90	56		
SCC: squamous cell carcinoma antigen; OS: overall survival; CEA: carcinoembryonic antigen; SUVmax: maximum standardized uptake value.				

### 2.3 SUVmax在Ⅰ期、Ⅱ期及Ⅲ期肺鳞癌患者中的预后分析

本研究中Ⅰ期病例共90例（49.4%），以SUVmax 13.0为界值将Ⅰ期肺鳞癌患者分为SUVmax高值组与SUVmax低值组，高值组预后差于低值组，差异具有统计学意义（*χ*^2^=3.681，*P*=0.045，图 2A）。Ⅱ期患者50例（27.5%），以SUVmax 13.0为界将其分为两组，进行生存分析发现SUVmax高值组与SUVmax低值组间的生存期不具统计学差异（*χ*^2^=0.120，*P*=0.729，图 2B）。Ⅲ期患者42例（23.1%），同样以SUVmax 13.0为界分为高值组与低值组，分析发现两组患者的生存期不具统计学差异（*χ*^2^=0.811，*P*=0.368，图 2C）。

**2:**
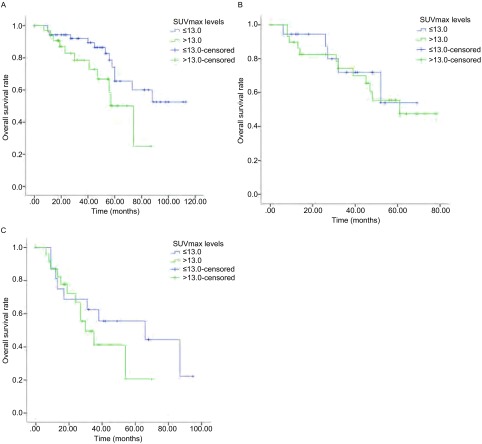
Ⅰ期（A）、Ⅱ期（B）和Ⅲ期（C）肺鳞癌患者中SUVmax分组与预后的关系 Relationship between SUVmax levels and prognosis in the stage Ⅰ (A), Ⅱ (B) and Ⅲ (C) lung squamous cell carcinoma

## 3 讨论

恶性肿瘤细胞分裂增殖异常活跃，与生长速度相比，肿瘤细胞所处环境能量相对匮乏，导致糖酵解增强，葡萄糖转运蛋白-1高表达，转运更多的葡萄糖以满足高代谢率和快速生长的需要，^18^FDG逐渐浓聚在恶性肿瘤细胞内，PET/CT用SUVmax来量化肿瘤对^18^FDG的摄取，从而获得肿瘤组织分子水平上的代谢活性信息^[10]^。正确认识原发灶SUVmax与肺癌临床病理因素及预后的关系，可以更好的指导临床工作。

肿瘤的发生发展与炎症反应密不可分^[11]^。NLR是血常规中性粒细胞绝对值与淋巴细胞绝对值之比，己成为反映全身炎症反应的简单易得且有效的指标。NLR升高即中性粒细胞增多及淋巴细胞减少，这打破了中性粒细胞与淋巴细胞之间原本的平衡状态。在肿瘤微环境中，中性粒细胞促进肿瘤细胞产生转化生长因子诱导肿瘤细胞发生上皮-间质转化，引发一系列信号转导和细胞骨架重塑，进而提高了肿瘤细胞的侵袭能力，最终导致肿瘤患者预后不良^[12]^。越来越多的证据^[13]^表明，无论组织内还是循环中的中性粒细胞均与包括肺癌、食管癌、肾癌、卵巢癌、结直肠癌在内的多种肿瘤患者的不良预后相关。原发灶SUVmax与中性粒细胞计数反映肿瘤环境糖酵解代谢增强及免疫失衡的特点，似乎可以解释SUVmax与中性粒细胞绝对值、NLR之间存在的正相关关系，本研究也发现中性粒细胞计数与不良预后有关（*P*=0.028），但未发现NLR与预后的关系（*P*=0.587）。

研究^[14]^证明肿瘤细胞可通过分泌白细胞介素-1，干扰素-*γ*和肿瘤坏死因子等可溶性细胞因子使红细胞发生溶血，抑制红细胞生成，并可降低促红细胞生成素的生理作用，从而导致贫血的发生。也有数据^[15]^表明乏氧环境能诱发基因突变，从而导致糖酵解增加，促进肿瘤血管的生成以抵御恶劣的生存环境。本研究发现原发灶SUVmax与血红蛋白呈负相关关系，可一定程度上反映出肿瘤对红细胞的抑制作用。

本研究中SUVmax与肿瘤最大径和TNM分期均呈正相关，与Zhang等^[16]^的研究结果一致，除去部分容积效应造成的假阴性差异，NSCLC原发灶大小是预测SUVmax的重要指标。Hubner等^[17]^解释SUVmax与原发灶大小的关系时提出SUVmax与肿瘤细胞增殖系数相关，病变早期的肿瘤病灶细胞数量少，增殖速度缓慢，对能量的需求少，因而对^18^FDG摄取低于较大的病灶，随着肿瘤体积的增大，细胞数量增多，需更多的糖代谢供能以维持肿瘤细胞的增殖、扩散，故^18^FDG的摄取增多，SUV增高。Stiles等^[18]^回顾性研究530例NSCLC（其中肺鳞癌占20%）患者中SUVmax与原发灶大小的关系，发现二者呈正相关，与本文研究结果一致。

多项研究表明SUVmax与NSCLC患者的不良预后显著相关。一项纳入21项研究的*meta*分析^[8]^显示较高SUV-max的NSCLC患者的生存差于SUVmax较低者。Bille等^[7]^以SUVmax中位数8.6为界分为两组研究413例NSCLC（肺鳞癌103例，占25.5%）手术患者原发灶SUVmax与预后的关系，多因素生存分析显示SUVmax（*P*=0.006）、TNM分期（*P* < 0.001）是影响肺癌患者术后生存的独立预后因素。Higashi等^[19]^对57例NSCLC患者进行分析，发现SUVmax > 5和SUVmax≤5的两组患者的术后5年生存率差异有统计学差异（*P* < 0.001），将单因素生存分析中有意义的影响因子（SUVmax、TNM分期）纳入*Cox*比例风险模型多因素分析证实只有SUVmax是影响预后的独立因素，提示SUVmax预测NSCLC患者预后的能力可能比TNM还要强。原发灶SUVmax判断预后所选用分界值的报道不尽一致，本研究以182例肺鳞癌原发灶SUVmax的中位数13.0为界分为两组，同样得到SUVmax高值组预后差于SUVmax低值组的结论（*P*=0.022），并且多因素分析显示SUVmax、TNM分期均为影响肺鳞癌术后患者生存的独立预后因素。以上研究表明SUVmax有独立于病理TNM分期之外的预后价值，并且本研究Ⅰ期患者中SUVmax高值组与低值组的生存之间的差异有统计学意义（*P*=0.045），说明用SUVmax对判断早期肺鳞癌患者的预后有一定作用，可将Ⅰ期患者按SUVmax的高低进一步分层以指导治疗及预后分析。同样对Ⅱ期及Ⅲ期患者应用相同的方法进行生存分析，均发现SUVmax高值组与低值组间生存期的差异无统计学意义，这可能与本研究中Ⅱ期及Ⅲ期患者样本量较少有关。目前已有一些研究^[20, 21]^报道了NSCLC中SUVmax在不同TNM分期中对生存的影响，研究结果各异，还未有其他研究专门以肺鳞癌患者为样本探讨SUVmax与预后的关系，还需更大的样本及长时间随访进一步研究分析。

手术是包括肺鳞癌在内的早期NSCLC患者治疗的首选，目前临床普遍认为Ⅰa期患者术后无需任何辅助治疗，但仍有约27%的患者于5年内死亡^[22]^。IALT（The International Adjuvant Lung Carcinoma Trial）研究发现Ⅰ期、Ⅱ期、Ⅲ期NSCLC均可从术后含顺铂类辅助化疗中获益，防止复发，但随访7.5年后化疗组会有更多死亡^[23, 24]^。所以临床亟需挑选出合适的目标患者开展最恰当的治疗以保证最大的临床获益。

原发灶SUVmax可直观地反映出肿瘤病灶的分子代谢水平，提供TNM分期以外的预后信息，所以若将相同的TNM分期患者区分为不同的预后危险组，可指导个体化治疗方案的制定。术前可根据SUVmax将Ⅰ期肺鳞癌患者分为预后高危组和低危组，术前预测预后较差，则术后可考虑行辅助治疗预防复发。

综上，PET/CT SUVmax对患者术后生存的预测有重要提示作用，是独立于TNM分期之外的一个重要预后因素，临床上可根据SUVmax的高低对同一TNM分期患者进行预后危险度分层，从而制定出更合适的个体化治疗方案以改善患者的预后。另外，原发灶SUVmax与肺鳞癌患者肿瘤大小、临床分期、NLR等临床因素相关，有助于加强PET/CT与临床病理因素间的联系，更好地服务于临床。
